# Seeking gene relationships in gene expression data using support vector machine regression

**DOI:** 10.1186/1753-6561-1-s1-s51

**Published:** 2007-12-18

**Authors:** Robert Yu, Kevin DeHoff, Christopher I Amos, Sanjay Shete

**Affiliations:** 1 Department of Epidemiology, Unit 1340, The University of Texas M. D. Anderson Cancer Center, 1155 Hermann Pressler Boulevard, Houston, Texas 77030, USA

## Abstract

Several genetic determinants responsible for individual variation in gene expression have been located using linkage and association analyses. These analyses have revealed regulatory relationships between genes. The heritability of expression variation as a quantitative phenotype reflects its underlying genetic architecture. Using support vector machine regression (SVMR) and gene ontological information, we proposed an approach to identify gene relationships in expression data provided by Genetic Analysis Workshop 15 that would facilitate subsequent genetic analyses. A group of related genes were selected for a shared biological theme, and SVMR was trained to form a regression model using the training gene expressions. The model was subsequently used to search for and capture similarly related genes. SVMR shows promising capability in modeling and seeking gene relationships through expression data.

## Background

In genome-wide linkage and association analyses using gene expression data from individuals in 14 CEPH (Centre d'Etude du Polymorphisme Humain) Utah families, Cheung and colleagues [1-3] found that variation in the expression level of the gene chitinase-3-like 2 (*CHI3L2*) was associated with a single-nucleotide polymorphism (SNP) marker, rs755467, in its promoter region. Other studies have also suggested that variation in a regulatory region of a gene is probably the main mediator of phenotypic divergence in evolution [4,5]. The expression variation pattern correlates with the genes' genetic architecture. The characteristics of expression variation patterns of genes in a biologically defined group may also describe the landscape of the genetic architectures of the genes. We proposed an approach to determine gene relationships on the basis of expression data so that the underlying genetic and biological classification can be established. Our approach will be useful in expression studies, which usually deal with thousands of genes.

## Methods

We adapted support vector machine regression (SVMR) [6,7] for our analysis because of its ability to handle multidimensional data and non-linear modeling. In SVMR formulation, the goal is to estimate an unknown continuous-value function based on a set of a finite number of samples (**x**_*i*_, *y*_*i*_), *i *= 1,..., *d *+ 1, where *d*-dimensional input **x **∈ R^*d *^and output *y *∈ R. In SVM regression, the input **x **is first mapped onto an *m*-dimensional feature space using a nonlinear mapping function called a kernel, and then a linear model is constructed in this feature space, which can be given by $f(z,\omega )=\sum_{j=1}^{m}\omega_{j}g_{j}(z)+b$, where *g*_*j*_(*z*), *j *= 1,..., *m *denotes a set of nonlinear transformations, and *b *is the "bias" term. The goal is to find an *f*(z, *ω*) that has at most *ε *deviation from the actually obtained target *y*_*i *_for all the training data and at the same time is as flat as possible to reduce the model complexity by minimizing the norm ||w||^2 ^in the following function:

$$\begin{array}{ccc} \min\frac{1}{2}||w|{|}^{2}+C\sum_{i=1}^{n}\left(\xi_{i}+\xi_{i}^{*}\right) & s.t. & \{\begin{array}{c} y_{i}-f(z_{i},\omega )\leq \varepsilon +\xi_{i}^{*}\\ f(z_{i},\omega )-y_{i}\leq \varepsilon +\xi_{i}^{*}\\ \xi_{i},\xi_{i}^{*}\geq 0,i=1,...,n \end{array}, \end{array}$$

where *ξ*_*i *_and $\xi_{i}^{*}$ are slack variables that define the "soft margin" to measure the deviation of training samples outside the *ε*-insensitive zone and *C *is the regularization parameter that determines the trade-off between model complexity (flatness) and the degree to which deviations larger than *ε *are tolerated in the optimization formulation.

We selected a certain number of genes in a defined relationship, e.g., sharing the same biological functions or protein family, as a training sample for SVMR with defined parameters (kernel functions, *ε*, and *C*), to learn their expression patterns. The learned SVMR was then used to "recruit" new expression data of another gene from outside the training set. With predefined criteria, SVMR judged whether the new gene belongs to the same group. The newly recruited genes then grew into a category of a relationship that is expected to show similarity with the defined relationship in the training set. Comparison of linkage analysis results, ontology information, and/or regulation pathways of the new genes with the existing ones will further evaluate the search results.

We devised a four-level search strategy to explore this approach: search within highly correlated genes, search within the genes of the same biological family, search using trained genes in one biological family over ones of another biological family, and "random walk" search using randomly picked genes to search randomly in the whole sample set (Table 1).

**Table 1:** SVMR 4-level search strategy and results

	Search level			
	
	1	2	3	4
Theme	Genes that contained highly correlated genes	From the same biological family	Across biological families	Random Walk (all genes)
Sample size	1000	55 RP^a ^49 ZFP^b^	49	3554
Sample selection criteria	A total of 1000 genes that contained 100 highly correlated genes	all in RP family, all in ZFP family	RP, ZFP, and DEAD^c^	The full data set of all 3554 genes
Training size	2 genes per training, 3 trainings	2 to 10 genes	3 genes per training	3 to 20 genes
Training selection criteria	Corr > 0.85, *p *< 0.001	Randomly from 55 RP genes or from 49 ZFP genes	Only from RP family	Randomly from entire sample
Best training size	2 genes	4–5 genes	3 genes	3–7 genes
Example of training genes	1. 200088_x_at and 200809_x_at (both are different problems for RPL12) (Pearson corr > 0.92 and Spearman corr > 0.90, *p *< 0.0001) 2. RPL32 and RPS18 (Pearson corr > 0.94, *p *< 0.0001) 3. DDX3Y and EIF1AY (Pearson corr > 0.9875, *p *< 0.0001)^d^	RPS11 RPS10 RPS3A (201257_x_at) RPS16	RPS4X RPS4Y1 RPS5	C1D ALOX5 ENO2 RERE
Example of captured genes	1. 200088_x_at and 200809_x_at 2. RPL32, RPS15, RPS18, RPS3A, and RPS28 3. DDX3Y and EIF1AY	1. RPL27, RPS3A(2000099_s_at), RPS3A(201257_x_at), RPS29, RPS28 2. RPS15A, RPS18, RPS12, RPS19 3. Similar results were seen among genes with ZFP family	DDX39 DDX3Y DDX58 DDX26	SCAP1 SGPP1 TGFBR3 CD9 VAMP8

^a^RP, ribosomal proteins family

^b^ZFP, zinc finger proteins family

^c^DEAD, DEAD box proteins, which are characterized by the conserved motif (Asp-Glu-Ala-Asp) (DEAD).

^d^Three pairs of highly correlated gene expressions as three separate training sets, and search separately back in the sample, and found itself and the others.

Study subjects were 194 individuals from 14 CEPH Utah families with 2819 genotyped SNPs across 22 autosomal chromosomes provided by Genetic Analysis Workshop 15. Expression data using 3554 gene probes in lymphoblastoid cells of the above subjects were obtained using Affymetrix Human Focus Arrays. Gene annotation and ontology information were available on 8793 genes, including the 3554 genes probed.

The gene expression data were tested for normality using Shapiro-Wilk and Anderson-Darling tests, and pair-wise correlations were tested using Pearson's correlation test for normally distributed expressions and Kendall and Spearman's test for non-normally distributed expressions. These tests were performed for all phenotypes that were stratified by generations in order to guide a better comparison in later relationship searches.

Quantitative trait locus (QTL) nonparametric linkage (NPL) linkage analyses were carried out using Merlin 1.0.1 for nonparametric QTL with options -qtl and -npl over the 2819 autosomal SNPs. This QTL NPL approach in Merlin provides nonparametric LOD score using quantitative trait-based on a general framework defined in the program's documentation [8]. We used the "1-Mb-to-1-cM" rule to convert the physical map into a genetic one. As a supplemental analysis, QTL regression analysis using Merlin-Regress was performed.

We broke down the given gene ontology information into minimum meaningful phrases and uploaded it into a database using mySQL4.1 for easy query. Genes of a biologically related group were selected using definitions in a database search, e.g., "ribosomal proteins" and "DNA repairing".

We used mySVM [6] for SVM regression. Once the training data were formed, the target data were assigned either randomly or in a predetermined manner, depending on the search scenario used. The predicted results were then compared with the observed values of the targeted gene, and the mean and standard deviations of the differences were calculated. The final rank of the results was based on both mean and standard deviation values. The lowest values ranked the highest, and usually the top 0.6–1.2% genes were selected as captured targets for further studies. Two types of kernel functions were used: dot and polynomial, with degree 1 through degree 4.

The biological relationship of the targeted genes to the training genes was inspected, comparing both genome-wide linkage results and ontological description and/or regulation pathways using *PathwayStudio *with ResNet 3.0 database (Ariadne Genomics, Inc.).

## Results

### Linkage analysis

Both non-parametric QTL linkage analysis and QTL regression linkage analysis were performed for selected genes on 22 autosomal chromosomes. Our results showed the NPL LOD scores ranged between 1 and 4.92, and the regression LOD scores fluctuated more dramatically, e.g., some LOD scores were >20.

**Figure 1:**
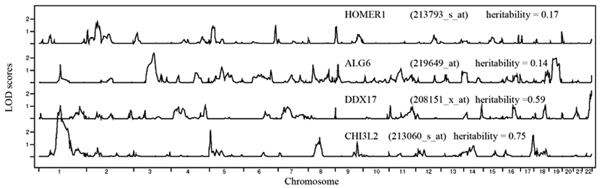
**Results of genome-wide linkage analysis of fourselected genes**. Linkage results for expressions of four genes that were compared with the ones presented in Morley et al. [2]. The Affymetrix probeset IDs are listed in parentheses.

We compared our QTL NPL results with those of Morley et al [2]. Of the 10 genes previously charted, we were able to find 9 in our 3554-gene expression data, and the comparison showed 5 of the 9, including *CHI3L2*, *DDX17*, and *ALG6*, were highly comparable in genome-wide LOD score distribution (Fig. 1). Three of the remaining four showed partial matches for distribution of LOD scores (either the relative peak height/width or location was slightly different). *HOMER1 *showed the least agreement with results from Morley et al.

### Searching among linear correlated genes

**Figure 2:**
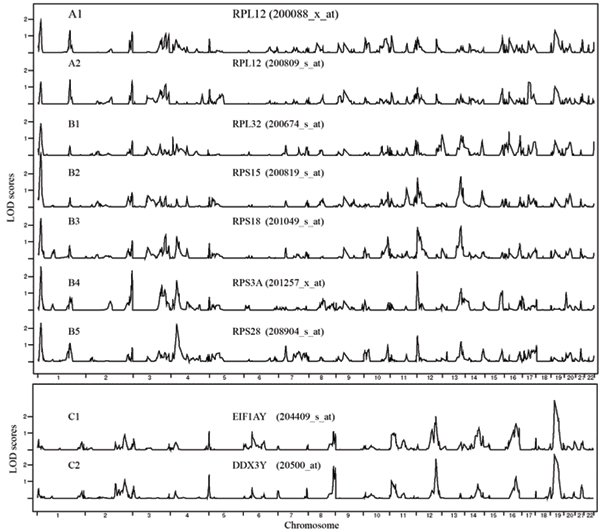
**Highly correlated expressions show similar linkage analysis results but may not be biologically related**. Expression data from groups A and B are highly correlated and they all belong to the same biological group, ribosomal proteins. A1 and A2 are from the same gene, *RPL12*. Correlation coefficients for expression of genes *C1 *and *C2 *are > 0.987, but they appear to share no direct biological relationship, even though their NPL LOD score distributions show high similarity as well.

We chose three pairs of highly correlated gene expressions as three separate training sets for SVMR and searched separately in a subset of 1000 genes that contained highly correlated genes. As we expected, the correlated expressions showed strong parallelism in their genome-wide LOD score distributions (Fig. 2 and Table 1).

### Searching among selected gene groups

We selected a group of genes from the same biological family, a set of 55 ribosomal protein genes. We found this group of genes closely shared similar biological functions, but not all were correlated in their expression data.

**Figure 3:**
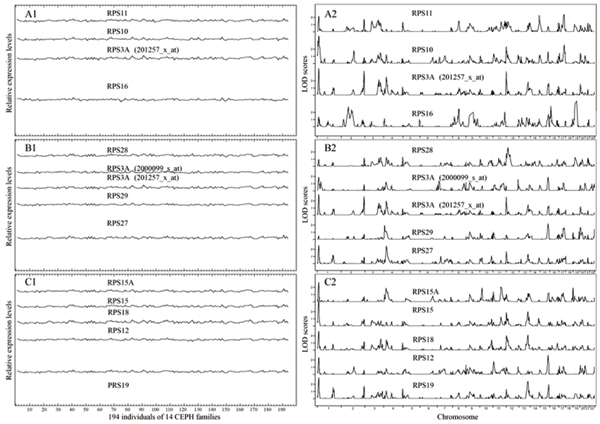
**SVMR training and searching results in one biological family – ribosomal proteins**. Expression pattern (left) and genome-wide NPL LOD score distributions (right) of ribosomal protein genes. Group A is the genes selected for the SVMR training set. Groups B and C are the genes that were targeted and captured in two separate SVMR searches.

We ranked the searched genes by the mean and standard deviation of the difference in expression levels between the selected gene and the one predicted from SVMR. If any search identified its own training genes, a high score for accuracy and specificity was given. Measurements of matching included both expression variation patterns and genome-wide LOD distribution (how many peaks in size and location along 22 chromosomes were matched or missed), and either a positive or negative point was scored for its sensitivity (Fig. 3 and Table 1).

We observed increasing specificity as the training set size grew, which seemed to taper off at a training set size of around seven genes. The sensitivity fluctuated slightly when the training set size was two or three but remained at over 96% when the size grew to four and above. A larger training set (with more than seven genes) may overfit the feature, causing difficulty in finding similar targets in the limited gene pool. A similar result was obtained in a zinc finger protein (ZFP) family (Table 1).

### Searching across gene groups

**Figure 4:**
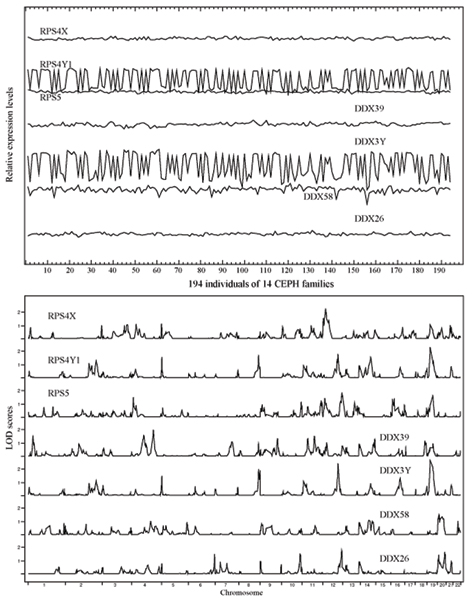
**SVMR searching results across biological families**. Expression pattern (top panel) and genome-wide NPL LOD score distribution of genes in training set (three ribosomal protein genes, *RPS4X*, *RPS4Y1*, *RPS5*) and the four captured DEAD box genes (*DDX39*, *DDX3Y*, *DDX58*, *DDX26*).

Using SVMR trained with only ribosomal protein genes, we searched a pool of another group of genes. Because of our limitations in time and computational resources, we made one attempt with a training set of three ribosomal protein genes (*RPS4X*, *RPS4Y1*, and *RPL18*), two being relatively "flat" in expression variation and one fluctuating more dramatically. We then formed two separate pools of targeting genes, one from the ZFP family and another composed of DEAD box protein genes, which are characterized by the conserved motif Asp-Glu-Ala-Asp. The results of this search strategy are given in Figure 4 and Table 1.

### Searching using the random walk method

We wanted to be able to discover a relationship directly, using gene expressions to form a training set and subsequently capturing a similar relationship. A random walk was designed to determine the size and makeup of a training set randomly and then to search a full set of gene expression samples randomly. This obviously required very heavy computational support. Therefore, we ran a short version of the plan and had a brief view of the random search outcome.

**Figure 5:**
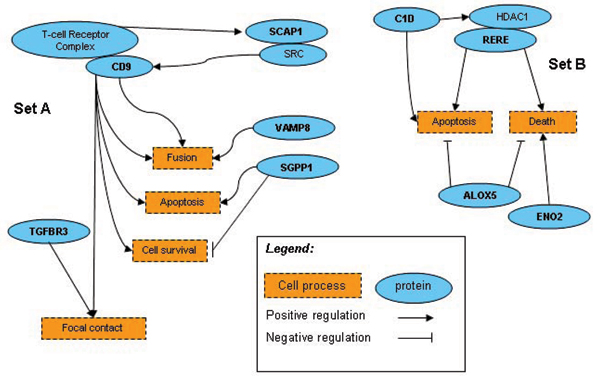
**Biological pathways of two groups of genes from "random walk" search**. Genes in set B were randomly picked for the SVMR training set, and then a random search of the gene pool hit a group of genes that formed set A. The pathway reconstruction was done using *PathwayStudio *4.0 (Ariadne Genomics, Inc.).

We randomly searched in the full set of expression samples. The total random walk was run in 30,000 rounds (one round equaled one training set with one set of randomly picked genes for prediction tests that was run once with each of the three kernels but scored together at the final stage). The top 1% scored targets were kept as candidates for further estimation of their biological relationships and/or genetic analysis. A randomly formed training set created a combination of genes with no pre-defined biological relationships. The expression variation patterns and linkage results were also different. Sometimes, matching expression data were overthrown by a contradictory linkage result and/or biological ontology description. This short version of random walk certainly only covered an infinitesimal fraction of the entire search space. In the 30,000 random runs, we only encountered one repetition of the same set of genes picked for the training set (but the testing set was different). However, we found two sets of genes, one from the training genes search and another from the subsequent search of the full set of expression samples, and each set had its connected regulation pathways (Fig. 5 and Table 1). Genes in set B that formed the training set, *C1D *(200056_s_at), *ALOX5 *(204446_s_at), *ENO2 *(201313_at), and *RERE *(200940_s_at), captured the ones in set A, *SCAP1 *(205790_at), *TGFBR3 *(204731_at), *SGPP1 *(221268_s_at), *CD9 *(201005_at), and *VAMP8 *(202546_at). Interestingly, the five genes in set A are all linked in the same region on chromosome 2, but set B doesn't have such characteristics (linkage results are not shown). How to relate the two sets of genes in terms of their biological connection or similarity remains to be further elucidated.

## Discussion

The pattern in gene expression variation does contain information that reflects the underlying genetic architecture. Using statistical learning machines like SVM can extend the capability to model more complex relationships with which regular statistical models such as regression may have limitations. In our exploration at four different searching levels, we noticed that the selection of genes for the training set, i.e., the definition of a biological relationship, influences the search results considerably. Meanwhile, the SNP composition and density, the heritability of expression data as a quantitative trait, and its distribution mode are major factors affecting both linkage results and SVMR learning quality.

We suggest that carefully processing expression data may help manage the data complexity, for example, through distinguishing heritability level, normality of phenotypic distribution, age stratification, or partitioning data using a defined theme to reduce noise level. But adding one or more dimensions of biological relationship information into the SVM learning process may increase the searching power by improving its specificity and sensitivity.

Our brief attempt at using the random walk method sheds light on the difficulty of discovering gene relationships directly via expression data. Genes in the same regulatory pathways share patterns of expression. Therefore, instead of searching an entire sample space, we plan to focus future research on adopting more effective search strategies such as those using genetic algorithms or other heuristic search approaches.

## Competing interests

The author(s) declare that they have no competing interests.
